# Age-dependent microstructure alterations in 5xFAD mice by high-resolution diffusion tensor imaging

**DOI:** 10.3389/fnins.2022.964654

**Published:** 2022-08-17

**Authors:** Surendra Maharjan, Andy P. Tsai, Peter B. Lin, Cynthia Ingraham, Megan R. Jewett, Gary E. Landreth, Adrian L. Oblak, Nian Wang

**Affiliations:** ^1^Department of Radiology and Imaging Sciences, Indiana University, Indianapolis, IN, United States; ^2^Stark Neurosciences Research Institute, Indiana University, Indianapolis, IN, United States; ^3^Department of Anatomy, Cell Biology and Physiology, Indiana University, Indianapolis, IN, United States

**Keywords:** Alzheimer’s disease, 5xFAD, MRI, DTI, diffusion MRI (dMRI)

## Abstract

**Purpose:**

To evaluate the age-dependent microstructure changes in 5xFAD mice using high-resolution diffusion tensor imaging (DTI).

**Methods:**

The 5xFAD mice at 4, 7.5, and 12 months and the wild-type controls at 4 months were scanned at 9.4T using a 3D echo-planar imaging (EPI) pulse sequence with the isotropic spatial resolution of 100 μm. The *b*-value was 3000 s/mm^2^ for all the diffusion MRI scans. The samples were also acquired with a gradient echo pulse sequence at 50 μm isotropic resolution. The microstructure changes were quantified with DTI metrics, including fractional anisotropy (FA) and mean diffusivity (MD). The conventional histology was performed to validate with MRI findings.

**Results:**

The FA values (*p* = 0.028) showed significant differences in the cortex between wild-type (WT) and 5xFAD mice at 4 months, while hippocampus, anterior commissure, corpus callosum, and fornix showed no significant differences for either FA and MD. FA values of 5xFAD mice gradually decreased in cortex (0.140 ± 0.007 at 4 months, 0.132 ± 0.008 at 7.5 months, 0.126 ± 0.013 at 12 months) and fornix (0.140 ± 0.007 at 4 months, 0.132 ± 0.008 at 7.5 months, 0.126 ± 0.013 at 12 months) with aging. Both FA (*p* = 0.029) and MD (*p* = 0.037) demonstrated significant differences in corpus callosum between 4 and 12 months age old. FA and MD were not significantly different in the hippocampus or anterior commissure. The age-dependent microstructure alterations were better captured by FA when compared to MD.

**Conclusion:**

FA showed higher sensitivity to monitor amyloid deposition in 5xFAD mice. DTI may be utilized as a sensitive biomarker to monitor beta-amyloid progression for preclinical studies.

## Introduction

Alzheimer’s disease (AD) is the most common cause of dementia with clinically characterized by age-dependent memory loss and cognitive dysfunction (Bush, 2003; Goedert and Spillantini, 2006). The primary characteristics of AD are the abnormal accumulation of extracellular β-amyloid (Aβ) and intracellular hyperphosphorylated tau proteins (Takahashi et al., 2010; Koss et al., 2016; Wang et al., 2016). Aβ plaque formation is considered as one of the earliest components, starting years, or even decades before clinical symptoms (Busciglio et al., 1995; Bloom, 2014). The capability to monitor the alterations in tissue microstructure caused by the accumulation of Aβ may provide an important biomarker to monitor disease progression or treatment response (Adlard et al., 2014; Thal et al., 2014).

Compared to the conventional volumetric MRI methods, diffusion MRI (dMRI) is more sensitive to the brain microstructural changes and has been widely used for the evaluation of white matter integrity (Bozzali et al., 2002; Mielke et al., 2009; Stebbins and Murphy, 2009; Wang et al., 2019b; Harrison et al., 2020; Veale et al., 2021). Patients with AD showed a significant reduction in the integrity of the associated white matter fiber tracts, including splenium of the corpus callosum, superior longitudinal fasciculus, and cingulum (Rose et al., 2000). Teipel et al. (2012) found a significant reduction of FA and a significant increase of MD in core areas of AD pathology including the corpus callosum, medial and temporal lobes, fornix, cingulate gyrus, precuneus, and prefrontal lobe white matter. The microstructure changes of patients with mild cognitive impairment have also been identified in the hippocampus using dMRI (Kantarci et al., 2005; Veale et al., 2021). More recently, dMRI studies have shown abnormalities in multiple neocortical areas at various stages of AD (Jacobs et al., 2013; Torso et al., 2021).

Despite the knowledge gained from human studies, transgenic animal models are an invaluable tool for studying pathogenic mechanisms and testing therapeutics of AD (Kitazawa et al., 2012; Jankowsky and Zheng, 2017; Sasaguri et al., 2017). The transgenic mouse model, 5xFAD, expresses human APP with three FAD mutations and human PSEN1 with two FAD mutations, and is commonly used to study the mechanisms of AD (Oakley et al., 2006; Forner et al., 2021). In 5xFAD mice, amyloid plaques are first observed between two and four months of age in the cortical layer V and in the subiculum of the hippocampal formation (Oblak et al., 2021). Different MRI techniques have been used to study the brain structure and function of 5xFAD mice (Mlynarik et al., 2012; Igarashi et al., 2020; Jullienne et al., 2022). Manganese-enhanced MRI (MEMRI) has been utilized as an activity-dependent contrast agent. Studies found that manganese could serve as a targeted contrast agent to visualize amyloid plaques in 5xFAD mice (Kim et al., 2021). The structural networks derived from dMRI exhibited higher path lengths in 6-month-old 5xFAD mice compared to controls (Kesler et al., 2018).

The 5xFAD transgenic mice have been acknowledged as a useful model for better understanding the pathogenesis of human AD (Girard et al., 2013). To the best of our knowledge, probing age-dependent tissue microstructure alterations in 5xFAD mice using high-resolution DTI has not been reported. In this study, we compared the microstructure alterations in cortex and hippocampus between wild-type (WT) and 5xFAD mice at 4 months of age. We further investigated the age-dependent microstructural variations in 5xFAD mice at three different ages (4, 7.5, and 12 months) throughout the whole brain (166 regions) (Wang et al., 2018a). The MRI findings were validated with Aβ plaque and NeuN staining.

## Materials and methods

### Animal preparation

Animal experiments were carried out in compliance with the Indiana University Institutional Animal Care and Use Committee. Six WT/six mice at the age of 4 months and eighteen 5xFAD (Jax #34848) mice at the ages of 4, 7.5, and 12 months (Jackson Laboratory, Bar Harbor, ME, United States) were sacrificed and perfused with the PBS solution. The mouse brains were immersed in buffered formalin for 24 h and then placed in a PBS solution of 0.5% Prohance (Bracco Diagnostics Inc., Princeton, NJ, United States) to shorten T1 and reduce scan time (Wang et al., 2019a).

### MRI protocol

MR images of the specimens were acquired on a 30-cm bore 9.4T magnet (Bruker BioSpec 94/30, Billerica, MA, United States) with a maximum gradient strength of 660 mT/m on each axis. A high-sensitivity cryogenic RF surface receive-only coil was used for signal reception (Bruker CryoProbe). A multi-shot 3D EPI pulse sequence was used with the following parameters: matrix size = 180 × 128 × 76, FOV = 18.0 mm × 12.8 mm × 7.6 mm, 100 μm isotropic spatial resolution, TE = 22.3 ms, TR = 100 ms, 61 unique diffusion directions with *b*-value of 3000 s/mm^2^ and six non-diffusion-weighted (b0) measurements (Crater et al., 2022). The gradient separation time was 4.4 ms and the diffusion gradient duration time was 9.8 ms.

A 3D gradient echo (GRE) pulse sequence was performed at the spatial resolution of 50 × 50 × 50 μm^3^ with TE of 12 ms (Wang et al., 2018b). The parameters were as follows: matrix size = 360 × 256 × 152, FOV = 18.0 mm × 12.8 mm × 7.6 mm, flip angle = 45°, bandwidth (BW) = 125 kHz, and TR = 100 ms.

### Data processing for diffusion tensor imaging

All the diffusion-weighted images (DWIs) were registered to the baseline images (b0) using linear affine registration. The scalar indices including FA, MD, axial diffusivity (AD), and

radial diffusivity (RD) were calculated based on the DTI model using DSI studio toolbox (Yeh et al., 2010). Each mouse brain was registered to the mouse brain atlas in Waxholm space using the Advanced Normalization Tools (ANTs) and then divided into 166 Region-of-interest (ROIs, Supplementary Figure 1; Wang et al., 2020a). The labels were mapped back to the individual brain space to calculate the values of DTI metrics in each ROI. A one-way analysis of variance (ANOVA) was performed to compare the statistically difference of DTI metrics between B6 and 5xFAD mice at the age of 4 months. To identify the age-dependent microstructure variations of 5xFAD mice, the DTI metrics were also compared at different ages: 4 months vs. 7.5 months, 4 months vs. 12 months. The statistical significance was determined at *p* < 0.05.

### Histology

Histological examinations were performed on the mice brains as previous described (Oblak et al., 2021; Tsai et al., 2021). Thirty micron-thick sections were stained to visualize neuronal cell bodies and beta-amyloid plaques using antibodies directed against NeuN (Abcam #ab104225, 1:1000, Boston, MA) and 6E10 (BioLegend #803001, 1:1000). The slides were imaged using Leica DVM6 digital microscope.

## Results

**FIGURE 1:**
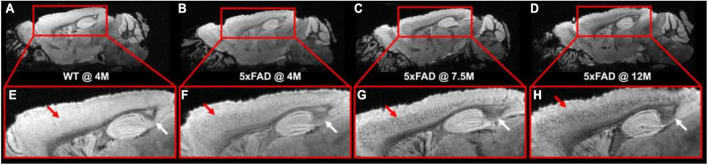
The T2*-weighted images of WT mice **(A,E)** at 4 months and 5xFAD at different ages (4, 7.5, and 12 months, **B–D**, **F–H**). Numerous dark spots were clearly evident in the cortex and hippocampus regions of the 5xFAD mice.

Figure 1 illustrates the T2*-weighted images of WT mice at 4 months and 5xFAD at different ages (4, 7.5, and 12 months, Figures 1B–D,F–H). As shown in the figure, numerous dark spots were evident in the cortex (red arrows) and hippocampus (white arrows) in 5xFAD mice. The hypointense signals were better observed under higher magnification, as displayed in the bottom row (Figures 1E–H). Such hypointense signals were not observed in the WT mouse (Figures 1A–E). The dark spot areas were gradually increased with age in the 5xFAD mice.

**FIGURE 2:**
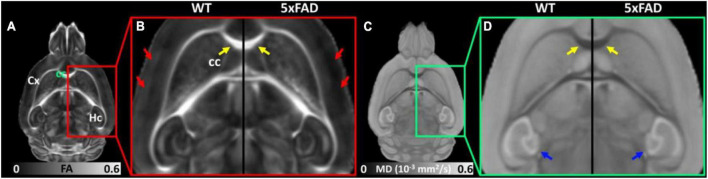
The representative FA **(A,B)** and MD **(C,D)** images of WT and 5xFAD mice at 4 months. There are no apparent FA and MD differences in the corpus callosum (cc, yellow arrows) and hippocampus (Hc, blue arrows) regions. The FA map showed darker (lower FA values) in the cortex (Cx) region of 5xFAD compared to WT (red arrows).

Figure 2 shows the representative FA (Figures 2A,B) and MD (Figures 2C,D) images of WT and 5xFAD mice at 4 months. Both WT and 5xFAD mice demonstrated very similar anatomy of the brain (Figure 2A). There are no apparent FA and MD differences in the corpus callosum (cc, yellow arrows) and hippocampus (Hc, blue arrows). The FA map showed darker (lower FA values) in the cortex (Cx) of 5xFAD compared to WT (red arrows). In contrast, the MD map depicted less variation between 5xFAD and WT mice.

**FIGURE 3:**
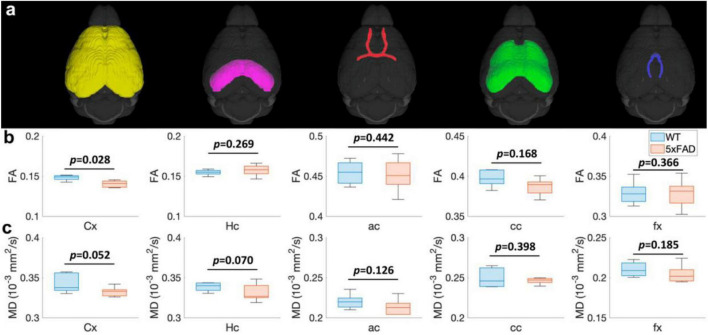
The FA **(B)** and MD **(C)** values at different regions (Cx, Hc, ac, cc, and fx, **A**) for both WT and 5xFAD mice at 4 months. Cx: cortex; Hc: hippocampus; ac: anterior commissure; cc: corpus callosum; fx: fornix.

The FA (Figure 3b) and MD (Figure 3c) values of different regions (Figure 3a) for both WT and 5xFAD mice at 4 months are shown in Figure 3. The different regions including Cx, Hc, anterior commissure (ac), cc, and fornix (fx) were rendered with different colors (Figure 3a). There were no significant differences in most of the brain regions, including Hc (*p* = 0.269 for FA; *p* = 0.007 for MD), ac (*p* = 0.442 for FA; *p* = 0.126 for MD), cc (*p* = 0.168 for FA; *p* = 0.398 for MD), and fx (*p* = 0.366 for FA; *p* = 0.185 for MD). In the cortex region, the FA exhibited a significant decrease between WT and 5xFAD mice (*p* = 0.028). In contrast, no significant difference was found for MD in the cortex region (*p* = 0.052).

**FIGURE 4:**
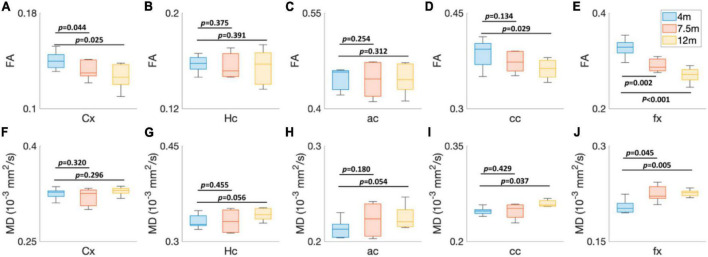
The age-dependent (4, 7.5, and 12 months) FA **(A–E)** and MD **(F–J)** values of 5xFAD mice at different brain regions. Cx, cortex; Hc, hippocampus; ac, anterior commissure; cc, corpus callosum; fx, fornix.

To explore the age-dependent tissue microstructure change in different brain regions, the FA and MD values of 5xFAD mice at different ages (4, 7.5, and 12 months) were exhibited in Figure 4. The DTI metrics showed strong age-dependent variations in different areas of the brain (Figures 4A–J). The FA and MD revealed no significant differences between 4 and 7.5 months in Hc (*p* = 0.375 for FA, *p* = 0.455 for MD), ac (*p* = 0.254 for FA, *p* = 0.180 for MD), and cc (*p* = 0.134 for FA and *p* = 0.429 for MD) regions. In contrast, both FA and MD showed significant differences in fx (*p* = 0.002 for FA and *p* = 0.045 for MD). FA values had significant differences in Cx (*p* = 0.044); MD values showed no significant differences in Cx (*p* = 0.320). More regions showed significant differences between 4 and 12 months, including Cx (*p* = 0.025 for FA), cc (*p* = 0.029 for FA and *p* = 0.037 for MD), and fx (*p* < 0.001 for FA and *p* = 0.005 for MD).

**FIGURE 5:**
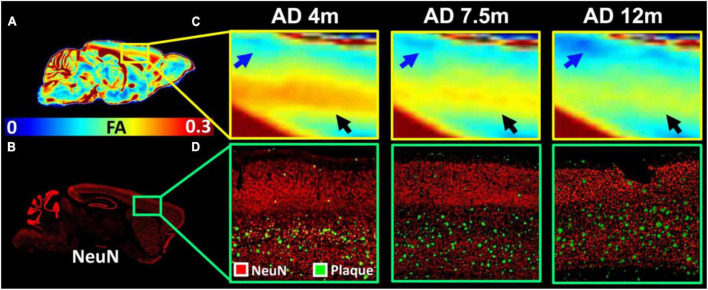
The age-dependent FA **(A,C)** and beta-amyloid staining images of 5xFAD mice **(B,D)**. The FA was gradually decreased with age in both upper layers (blue arrows in **C**) and lower layers (black arrows in **C**) of the cortex. The beta-amyloid staining was overlayed on the NeuN staining **(D)**.

To validate the MRI findings with conventional histology, the age-dependent FA (Figures 5A,C) and beta-amyloid staining (Figures 5B,D) images of 5xFAD mice are shown in Figure 5. The FA was gradually decreased with age in both upper layers (blue arrows in Figure 5C) and lower layers (black arrows in Figure 5C) of the cortex. The beta-amyloid staining was overlayed on the NeuN staining (Figure 5D). There were more plaques in the lower layers of the cortex regardless of the age. The plaques gradually increased with age in both lower and upper layers of the cortex. The representative FA slices at different ages (4, 7.5, and 12 m) are shown in Supplementary Figure 2.

**FIGURE 6:**
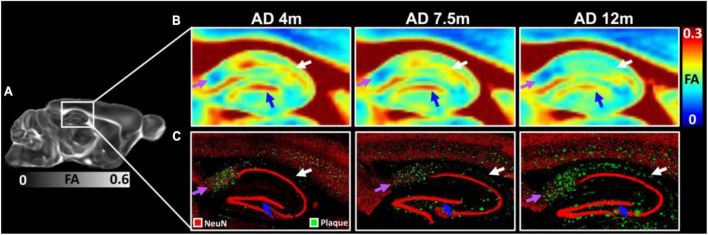
The FA images **(A,B)** and beta-amyloid staining **(C)** at different ages of 5xFAD in hippocampus region. The FA values showed strongly age-dependent in the dentate gyrus (DG, blue arrows), the dorsal subiculum (DS, purple arrows), and the CA2 (white arrows). The plaques gradually increased with age in the hippocampus regions **(C)**.

The FA images (Figures 6A,B) and beta-amyloid staining (Figure 6C) at different ages of 5xFAD in the hippocampus are illustrated in Figure 6. The FA values showed strong age-dependent changes in the dentate gyrus (DG, blue arrows in Figure 6B), the dorsal subiculum (DS, purple arrows in Figure 6B), and the CA2 (white arrows). The plaques gradually increased with age in the hippocampus regions (Figure 6C). There were more plaques accumulated in the DG, DS, and CA2 regions, which was consistent with the reduction of FA.

**FIGURE 7:**
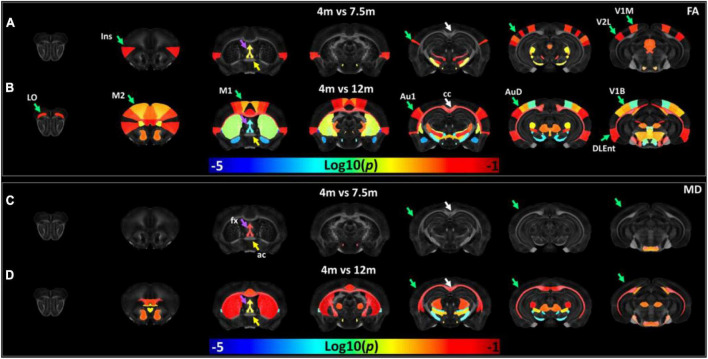
The age-dependent whole-brain microstructure changes in 5xFAD mice. The fractional anisotropy (FA) statistic maps (**A,B**) exhibited higher sensitivity to the age effect compared to mean diffusivity (MD) (**C,D**). Ins, insular cortex; V1M, primary visual cortex monocular area; V2L, secondary visual cortex lateral area; fx, fornix; M1, primary motor cortex; M2, secondary motor cortex; LO, lateral orbital cortex; Au1, primary auditory cortex; AuD, secondary auditory cortex dorsal part; V1B, primary visual cortex binocular area; DIEnt, dorsal intermediate entorhinal cortex; cc, corpus callosum; Cpu, striatum.

The age-dependent whole-brain microstructure changes in 5xFAD mice were demonstrated in Figure 7. In general, FA exhibited higher sensitivity to the age effect than MD. The cortex regions including the insular cortex (Ins), primary visual cortex monocular area (V1M), secondary visual cortex lateral area (V2L), primary auditory cortex (Au1), and fornix showed significant differences in the FA statistical maps (4 vs. 7.5 months). There were more regions with significant differences between 4 and 12 months, including the primary motor cortex (M1), secondary motor cortex (M2), lateral orbital cortex (LO), secondary auditory cortex dorsal part (AuD), primary visual cortex binocular area (V1B), dorsal intermediate entorhinal cortex (DIEnt), corpus callosum (cc), and striatum (Cpu). Compared to the FA statistical maps, the MD statistical map showed fewer age-dependent alterations (4 vs. 7.5 months, 4 vs. 12 months). MD showed no significant differences in the cortex regions between 5xFAD at 4 and 7.5 months, MD showed significant differences in Cpu and cc between 5xFAD at 4 and 12 months. The fx showed significant differences in both comparisons (4 vs. 7.5 months, 4 vs. 12 months), while ac showed no significant differences among different ages.

## Discussion

Diffusion tensor imaging is the most widely used dMRI technique to access neurodegenerative and pathophysiology diseases (Kantarci et al., 2010; Teipel et al., 2012). TDTI metrics including fractional anisotropy (FA) and mean diffusivity (MD) have been used as potential imaging biomarkers for the diagnosis of AD (Teipel et al., 2011; Nir et al., 2013). The 5xFAD is one of the most common mouse models to study the beta-amyloid aspects of human AD, its microstructure change with age has not been investigated in detail (Oakley et al., 2006). In this study, we acquired whole-brain high-angular resolution dMRI at 100 μm^3^ and investigated the age-dependent (4, 7.5, and 12 months) DTI metrics variations in different regions of the brain (166 ROIs).

The beta-amyloid plaques are a hallmark of AD that develop in its early stage (Reger et al., 2008). Non-invasive detection of these plaques would be invaluable for diagnosis and monitoring the treatment. The MRI signal has been enhanced by the MEMRI to visualize the Aβ plaques in transgenic rodent models of AD (Kim et al., 2021). Dudeffant et al. (2017) demonstrated that Gadolinium (Gd)-stained MRI can be used to detect the amyloid plaques in both mouse AD models and human-AD brains. The Aβ plaques could also be detected in mouse AD models using a high-resolution T2-weighted spin-echo sequence (Jack et al., 2005). In this study, high-resolution GRE pulse sequence (50 μm isotropic) was applied to detect the AB plaques. The plaques showed hypointensity in MRI images and increased with age in both cortex and hippocampus regions, which is consistent with several previous findings (Higuchi et al., 2005; Meadowcroft et al., 2009). Distinguishing individual amyloid plaques by high-resolution MRI might provide a noninvasive estimate of plaque burden in transgenic AD mice that might be useful in assessing the efficacy of anti-amyloid therapies (Santin et al., 2016).

Several studies have demonstrated that DTI changes precede the anatomical changes on structural MRI for detecting the brain impairment in different mice models (Zhang et al., 2012; Muller et al., 2020). However, the observed DTI scalars are not consistent across studies, probably due to the dynamic microstructural change in different animal models. For instance, FA in the cortex was reported to increase in 3xTg AD mice (Snow et al., 2017; Manno et al., 2019), while the FA was showed reduced in APP transgenic mice (Muller et al., 2013; Qin et al., 2013). It’s also suggested that DTI studies of AD models with marked neuron loss indicate increased MD, whereas those of a mild neuropathological phenotype without neuron loss tend to report decreased MD (Snow et al., 2017). Compared to MD, we demonstrated the reduction of FA in the cortex region can be detected as early as 4 months between WT and 5xFAD. The cortex area exhibited lower FA values and higher plaque loading in 5xFAD mice with age, which suggests that FA could be a sensitive imaging biomarker to detect the beta-amyloid pathology in the AD mice.

In the current study, we divided the whole brain into 166 different regions, in which more than 40 ROIs belong to isocortex (Wang et al., 2020a). It allows us to investigate the spatial–temporal pattern of the microstructure with the beta-amyloid progression in a well-established AD mouse model. At 7.5 months age, primary visual cortex and secondary visual cortex have already reduced the FA values significantly. At 12 months age, more cortex regions exhibited microstructure alterations, including the primary visual cortex, secondary auditory cortex, primary auditory cortex, dorsolateral entorhinal cortex, and motor cortex. In contrast, fewer cortex regions showed significant MD changes compared to FA values, which suggests that FA could be used to detect the inhomogeneous progression of beta-amyloid deposition at different regions of the cortex area.

Similar to the cortex, amyloid plaque loading is known to be high in hippocampus (Lazarov et al., 2002; Vyas et al., 2020). Reduced FA and increased MD in hippocampus were reported in many of these human studies, probably attributed to increased extracellular space volume and neurodegeneration (Muller et al., 2007; Cherubini et al., 2010; Hong et al., 2013; Nowrangi et al., 2013). It has been reported that only FA showed a significant increase in the hippocampus of APP/PS1 mice, compared to other DTI metrics (Shu et al., 2013). In contrast, decreased FA was detected in the hippocampus in the 12-14-month-old 3xTG AD mice (Snow et al., 2017). In the current study, both FA and MD showed no significant differences for the ROI-based analysis (whole hippocampus). The inconsistency may cause by different mouse models and different ages of mice in different studies. The heterogeneous FA alterations were observed in the subregions of the hippocampus, which may be associated primarily with the plaque deposition and related inflammation (Praet et al., 2018). It has been noted that the hippocampus exhibits complex microstructure and has been demonstrated to show laminar features due to layer-dependent FA values (Wang et al., 2020b). The higher spatial resolution dMRI with sub-region analysis should be performed in future studies to investigate the inhomogeneous microstructure alterations at different layers of the hippocampus (Wu et al., 2020).

Alzheimer’s disease is classically considered a disease of gray matter; however, white matter abnormalities have been widely reported in the brains of incipient and mildly afflicted individuals (Sachdev et al., 2013). Advanced neuroimaging studies demonstrate that patients with preclinical AD have widespread white matter abnormalities at a stage similar to those reported in AD (Marquez and Yassa, 2019; Stone et al., 2021). White matter abnormalities, particularly axonal transport deficiencies, are also important components of AD (Klok et al., 2018). The fornix is a white matter bundle belonging to the medial diencephalon and serves a vital role in memory functions (Copenhaver et al., 2006; Nowrangi and Rosenberg, 2015). Fornix microstructural degradation, as measured by reduced FA, was prominent in both MCI and AD, and may provide evidence of degenerative white matter injury in AD (Lee et al., 2012). The age-dependent microstructural alterations of fx were demonstrated by both FA and MD in 5xFAD mice. In contrast, the ac showed no significant changes among different ages (4 m–12 m), which is consistent with a recent study using the P301L mouse model (Massalimova et al., 2021). Compared to other white matter bundles, fornix may play an important role in AD pathology and can be used as a disease biomarker for pharmacological therapeutics (Badea et al., 2016).

There are several limitations in our study. First, we only performed ROI-based analysis due to the limited sample size, the voxel-based analysis should be performed with larger sample size. Second, compared to *in vivo* studies, *ex vivo* MRI affords higher resolution and fewer motion artifacts, it may not accurately represent the tissue microstructures under normal physiological conditions due to the fixation. Third, only female 5xFAD mice were conducted in the study, the sex difference effect is warranted in future studies. In addition, transgenic models have been widely used to study AD risk factors and mechanisms, but the degree of characteristics displayed in comparison with AD in humans is still limited (Elder et al., 2010).

## Conclusion

We demonstrated that microstructure alterations are age-dependent using a model of progressive brain amyloidosis. We were able to detect the inhomogeneous FA alterations in different parts of cortex and hippocampus. The spatial–temporal pattern of the microstructure changes in the cortex region with the beta-amyloid progression has been generated. DTI metrics could be used to monitor the progression of beta-amyloid deposition and the efficiency of the anti-AD drugs.

## Data availability statement

The original contributions presented in this study are included in the article/Supplementary material, further inquiries can be directed to the corresponding author.

## Ethics statement

The animal study was reviewed and approved by Indiana University Institutional Animal Care and Use Committee.

## Author contributions

SM and NW: study conception and design and draft manuscript preparation. AT, PL, CI, MJ, and NW: data collection. SM, GL, AO, and NW: analysis and interpretation of results. All authors reviewed the manuscript and approved the submitted version.
