# Corrigendum: Age-dependent microstructure alterations in 5xFAD mice by high-resolution diffusion tensor imaging

**DOI:** 10.3389/fnins.2022.1025457

**Published:** 2022-10-13

**Authors:** Surendra Maharjan, Andy P. Tsai, Peter B. Lin, Cynthia Ingraham, Megan R. Jewett, Gary E. Landreth, Adrian L. Oblak, Nian Wang

**Affiliations:** ^1^Department of Radiology and Imaging Sciences, Indiana University, Indianapolis, IN, United States; ^2^Stark Neurosciences Research Institute, Indiana University, Indianapolis, IN, United States; ^3^Department of Anatomy, Cell Biology and Physiology, Indiana University, Indianapolis, IN, United States

**Keywords:** Alzheimer's disease, 5xFAD, MRI, DTI, diffusion MRI (dMRI)

In the published article, there was an error in **Materials and methods**, “*Histology*,” paragraph 1. The incorrect histology protocol was used and the description of the histology (slice thickness, antibody of NeuN, microscope) was therefore incorrect. The paragraph previously stated:

“Histological examinations were performed on the mice brains as previous described (Oblak et al., 2021; Tsai et al., 2021). Coronal 8-μm thick slices were stained immunocytochemically stained for the neuronal nuclear antigen (NeuN) (MAB377, lot 2967854, Millipore, Burlington, MA, United States) and 6E10 staining (BioLegend #803001 in mouse, 1:1000; AB_2564653) for beta-amyloid plaques. The slides were imaged using Axioscop2 FSmot optical microscope with EC PlanNeofluar Zeiss lens at 20 × magnification, 0.3 aperture under the same settings and light conditions.”

The corrected paragraph appears below:

“Histological examinations were performed on the mice brains as previous described (Oblak et al., 2021; Tsai et al., 2021). Thirty micron-thick sections were stained to visualize neuronal cell bodies and beta-amyloid plaques using antibodies directed against NeuN (Abcam #ab104225, 1:1000, Boston, MA) and 6E10 (BioLegend #803001, 1:1000). The slides were imaged using Leica DVM6 digital microscope.”

The authors apologize for this error and state that this does not change the scientific conclusions of the article in any way. The original article has been updated.

## Publisher's note

All claims expressed in this article are solely those of the authors and do not necessarily represent those of their affiliated organizations, or those of the publisher, the editors and the reviewers. Any product that may be evaluated in this article, or claim that may be made by its manufacturer, is not guaranteed or endorsed by the publisher.
