# Supplementary material for: Age-dependent microstructure alterations in 5xFAD mice by high-resolution diffusion tensor imaging
Source: Front Neurosci. 2022 Aug 17;16:964654. doi: 10.3389/fnins.2022.964654 (PMC9428354; doi:10.3389/fnins.2022.964654)
Supplement: Supplementary file 1 [file Image_1.pdf]

Supporting Information

Additional supporting information may be found online in the Supporting Information section at the end of the article.

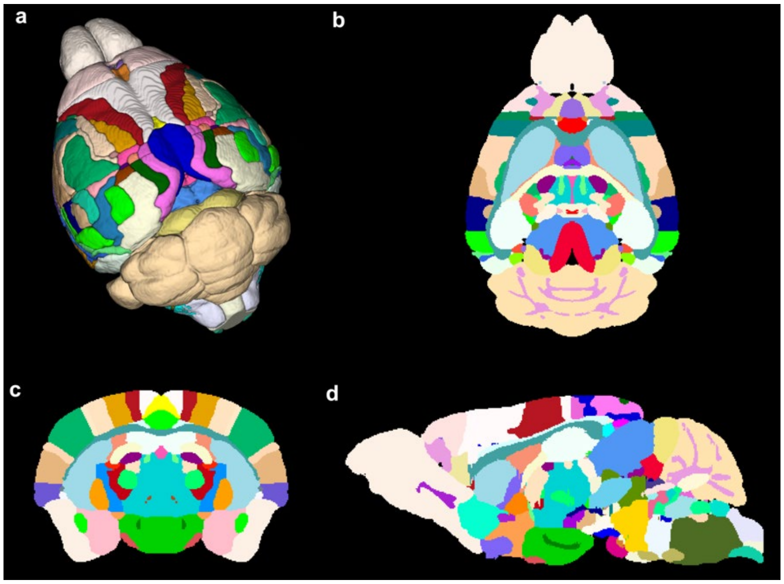

Supporting Information Figure S1. The parcellation of the mouse brain into 166 ROIs.

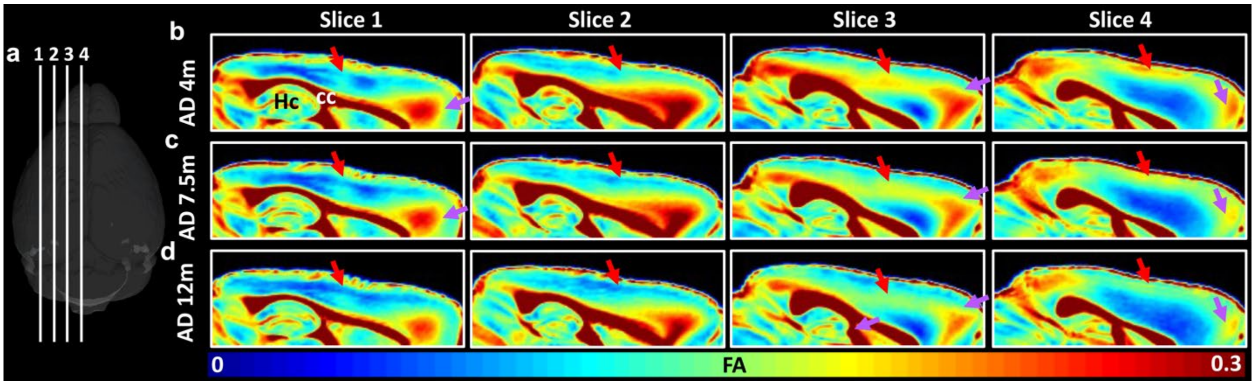

Supporting Information Figure S2. The representative slices showing the FA changes with age (4, 7.5, and 12 months) at cortex regions in 5xFAD mice.
